# Durable Complete Radiological Response to Nivolumab in Two Heavily Pretreated Western Elderly Patients With Metastatic Gastric Cancer: A Case Report

**DOI:** 10.3389/fonc.2020.00130

**Published:** 2020-02-17

**Authors:** Giuseppe Tirino, Angelica Petrillo, Luca Pompella, Annalisa Pappalardo, Maria Maddalena Laterza, Iacopo Panarese, Rosalaura Sabetta, Renato Franco, Gennaro Galizia, Fortunato Ciardiello, Ferdinando De Vita

**Affiliations:** ^1^Department of Precision Medicine, University of Campania Luigi Vanvitelli, Naples, Italy; ^2^Dipartimento di Salute Mentale e Fisica e Medicina Preventiva, Università degli Studi della Campania Luigi Vanvitelli, Naples, Italy; ^3^Department of Translational Medical Sciences, University of Campania Luigi Vanvitelli, Naples, Italy

**Keywords:** gastric/GEJ adenocarcinoma, immunotherapy, nivolumab, checkpoint inhibition, gastrointestinal cancer

## Abstract

**Background:** The prognosis of patients with advanced gastric cancer remains overall poor despite some recent innovations and the development of new therapeutic approaches. Current European guidelines do not recommend any specific treatment for patients with advanced gastric cancer refractory to two or more previous chemotherapy regimens, making this setting “orphan.” Immunotherapy is quickly evolving also for this malignancy even if with controversial results and the correct patient selection is still debated, especially for Western patients. The phase III ONO-4538-12 “ATTRACTION-2” represents the current landmark trial for the development of immunotherapy for pretreated Asian patients and led to the approval of Nivolumab in some Asian countries, while only previous phase trials are available for Caucasians. Complete radiological response is anecdotic and has never been described both in the pivotal trial both in the others with Western patients enrolled.

**Case presentation:** We report two cases of heavily pretreated Western elderly patients with metastatic gastric cancer who experienced durable complete radiological response to Nivolumab “off label” (more than 20 months to date) in a clinical practice context. Molecular analysis of potential predictive factors has been performed (PD-L1, EBV, MSI, and TMB) on primary tumor sample.

**Conclusions:** Despite the lack of evidence for Western patients and the controversial outcome with the use of checkpoint inhibitors in previous settings, immunotherapy may dramatically change the prognosis and the natural history of pretreated Western metastatic gastric cancer, in a correctly selected population. Microsatellite instability and tumor mutational burden may be reliable predictive factors also for Caucasians. There is an urgent need for a change in clinical practice also for this “orphan” patients and more efforts are needed in order to clarify the role of predictive factors for a correct patient selection and better chances of survival for this awful malignancy.

## Background

The prognosis of patients with advanced gastric cancer (GC) remains poor and the median overall survival is still around 1 year despite some recent innovations and the development of new therapeutic approaches (1, 2).

Current European guidelines do not recommend any specific treatment for patients with disease refractory to two or more previous regimens. Anyway, the amount of patients who reach this setting is increasing and nowadays only some Asian countries and the US registered immunotherapy for advanced GC in this specific population (3–5).

Immunotherapy is quickly evolving also for GC even if it is not yet a standard of care worldwide and the results are controversial. Furthermore, the correct patient selection remains a target not fully achieved, especially for Western patients.

For Asian patients the phase III trial ONO-4538-12 “ATTRACTION-2” (6) represents the current milestone for the development of immunotherapy with Nivolumab (anti-PD-1 antibody) in the chemotherapy-refractory molecularly unselected population, while evidence for Western population is limited to early phase trials (7, 8). In this Asian trial surprising survival rates of 27.3 and 10.6% at 1- and 2 year, respectively, have been achieved in the Nivolumab arm (with a 86.7% 12 months survival rate in responders), suggesting the efficacy of immunotherapy and the presence of a subset of patients who greatly benefit from it even if molecular features of responders are still not well-understood.

Moreover, in first and second line setting immunotherapy did not significantly improve survival compared to chemotherapy both in Asian and Western patients in recent phase III randomized trials KEYNOTE-062 (9) and KEYNOTE-061 (10).

With regards to biomarkers selection, a recent prospective phase II trial with Pembrolizumab (anti-PD-1 antibody) (11) clarified the positive predictive role of the Epstein-Barr Virus (EBV) and microsatellite instability status (MSI), but, once again, the population was entirely from Asia.

The same molecular features (Mismatch repair and EBV status), also with PD-L1 expression and tumor mutational burden (TMB), have been recently analyzed in a single institution Japanese study with 80 patients treated with Nivolumab after two or more chemotherapy regimens (12). These markers were strongly associated with favorable response to treatment and might represent useful selective and predictive factors.

However, there are no results regarding the subgroup analysis according to MSI and programmed death-ligand 1 (PD-L1) status in the ATTRACTION-2 population.

Genetic, epidemiologic, and molecular differences between Asian and western patients are well-known. Even if data from clinical trials are lacking for non-Asians, we should consider that there is some evidence that Western patients seem to have a more favorable “immune-environment” and a stronger immune-signature. In fact, a large global retrospective study with 1,600 GC samples from both Asian and non-Asian cohorts showed different tumor immune-signatures between Asian and non-Asian patients, particularly with infiltrating T-cells and T-cell gene expression patterns in Caucasians that might condition a possible more frequent and better response to immunotherapy (13, 14).

Here we report two cases of elderly pretreated Western patients with metastatic GC who have experienced extraordinary and dramatic durable response with “*off label”* Nivolumab in a clinical practice context.

In particular, we report the first radiologic complete responses to Nivolumab in Western patients with advanced pretreated GC at our knowledge, highlighting that immunotherapy is not a standard of care for GC and complete responses are anecdotal at current time and still not described even in the landmark phase III trial such as in the other mentioned studies with Western patients enrolled (with the exception of a single possible complete response registered in the monotherapy arm of the phase II Checkmate-032, not confirmed by the independent central review).

## Materials and Methods

Radiological evaluation for advanced disease has been performed every 3 or 4 months approximately using total body computed tomography (CT) with contrast with consecutive comparisons and response assessment performed according to response evaluation criteria in solid tumors (RECIST) v1.1 (15). A 18- fluorodeoxyglucose positron emission tomography (18F-FDG PET) and magnetic resonance imaging (MRI) with contrast have been used as second level imaging in order to deepen radiological findings when clinically indicated.

Treatment toxicity has been evaluated according to Common Terminology Criteria for Adverse Events (CTCAE) v5.0 (16).

Histological and molecular analysis have been performed on the surgical sample of primary tumor in local laboratory according to current guidelines, with the exception of “FoundationOne CDx” test (a next generation sequencing based comprehensive genomic profiling offered to patients free of charge at our institution as companion diagnostic) (17).

Immunohistochemical evaluation of mismatch repair (MMR) proteins status (MLH1, MSH2, PMS2, and MSH6) and PD-L1 expression (tumor proportion score), Epstein-Barr early RNA (EBER) *in situ* hybridization for EBV, in addition to human epidermal growth factor receptor 2 (HER2) status evaluation, were performed in local laboratory according to the European Society of Medical Oncology recommendations (Table S1).

Tumors lacking either MLH1, MSH2, PMS2, or MSH6 expression were considered “MMR-deficient,” while MSI and TMB have been assessed only by FoundationOne CDx.

At the moment of disease progression after the second or third line of treatment, patients signed the informed consent and an off label use request for Nivolumab was submitted for each patient in light of the results of phase III Asian trial “ATTRACTION-2” presented at ASCO GI 2017 and ESMO congress 2017. It is noteworthy that current guidelines did not recommend any specific treatment for this specific setting. Nivolumab has been administered 3 mg/Kg iv in a 14 days cycle until progression of disease or unacceptable toxicity. Every biweekly administration has been considered a cycle of treatment.

Local ethical committee has been regularly updated about this off label program and a specific informed consent was obtained from both patients for the publication of this report.

The survival and safety follow up cut-off date for this report was June 2019, 20th.

## Case Presentation

### Patient 1

**Figure 1:**
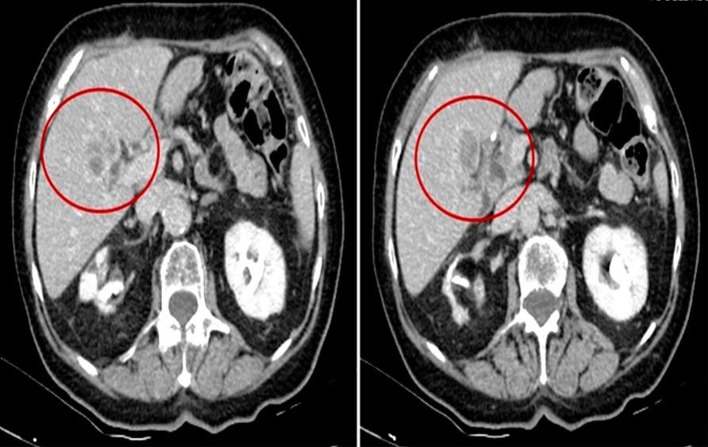
Basal 28 mm liver metastasis at IV segment in 2015/2016 (patient 1).

Patient 1, a Caucasian 78 year-old female, has been diagnosed on February 2015 with a well-differentiated adenocarcinoma of the body of the stomach, intestinal type according to Lauren classification, with staging CT scan evidence of liver metastasis (28 mm at IV segment) (Figure 1). She was in good clinical condition (ECOG performance status 1); blood hypertension, hypothyroidism, arthrosis, and stage 1 chronic kidney disease with “contracted kidney” co-occurred at the moment of diagnosis. She was complaining of dyspepsia and slight unintentional weight loss since few weeks.

In March 2015, she underwent a palliative total gastrectomy with roux-en-y esophagojejunostomy and D2 lymphadenectomy due to symptomatic luminal obstruction. The stage according to TNM was pT4 G1 pN0 (0/26) cM1 HER2 negative.

From April 2015 to September 2015 she received 8 cycles of mFOLFOX-6 as first line chemotherapy, achieving a stable disease as best objective response, and subsequently continued maintenance treatment with capecitabine single agent in light of the side effects and the age until December 2015, when she was referred for follow-up due to worsening skin and GI toxicity.

On September 2016 (PFS = 16 months), a liver progression occurred (same known lesion of 47 mm, ECOG performance status 1 confirmed) and patient started the second line treatment with Ramucirumab 8 mg/kg (d1,15) plus Paclitaxel 80 mg/mq (d1,8,15) administered in a 28 days cycle (18).

After 6 cycles, she obtained a partial response and continued Ramucirumab monotherapy until May 2017 due to grade 3 neurotoxicity.

In May 2017, patient underwent a hip bone and femurs MRI with contrast in order to evaluate a new onset skeletal pain. MRI showed a secondary osteolytic lesion at right femur (Figure S1) and the CT scan showed a slight progression of the known liver lesion in absence of new ones.

Therefore, in June 2017 patient was referred for antalgic radiotherapy (8 Gray single fraction) and antiresorptive therapy with zoledronic acid iv. Moreover, she refused radiation therapy for liver lesion and further chemotherapy also considering age and the toxicity of previous chemotherapy regimens.

After 3 months of follow up, the off label immunotherapy with Nivolumab was available and patient started treatment on 25 September 2017.

In January 2018, the first CT and PET evaluation demonstrated a stable disease according to RECIST criteria. No weight loss, skeletal pain, or other symptoms occurred. Meanwhile, patient experienced a global clinical condition improvement and a significant recovery from previous chemotherapy toxicity. No immune-related adverse events were recorded.

In June 2018, she experienced a slight deterioration of renal function (likely related to zoledronic acid use) with a serum creatinine of 2 mg/dl and ultrasound evidence of monolateral grade 1 hydroureteronephrosis and concurrent lithiasis. A CT scan without contrast confirmed the nephrological findings and no more evidence of liver disease (as already shown at abdominal ultrasound) as well as no potential signs or peritoneal involvement.

Zoledronic acid was discontinued and immunotherapy temporarily interrupted due to the persistence of creatinine increase. In July 2018, patient underwent ureteral stent placement (subsequently removed in October 2018 due to recurrent urinary tract infections) and creatinine returned to baseline levels during the next weeks.

**Figure 2:**
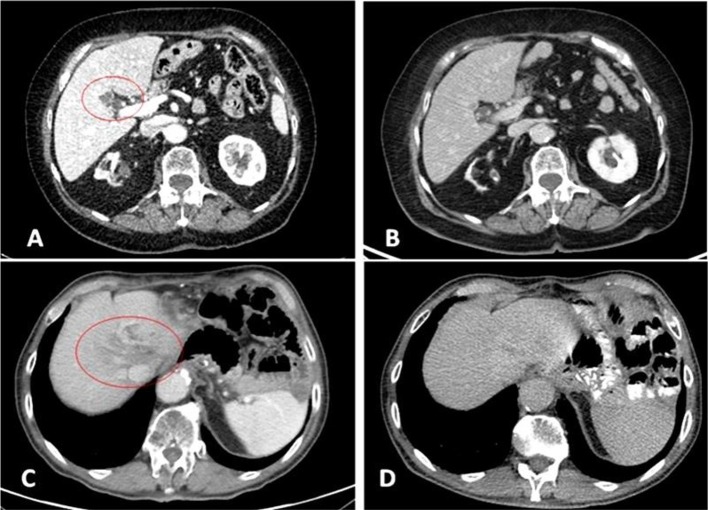
2017 pre-treatment (**A** = patient 1; **C** = patient 2) and 2019 complete response CT scans (**B** = patient 1; **D** = patient 2). Liver metastases at IV **(A)** and VIII **(C)** segment.

**Figure 3:**
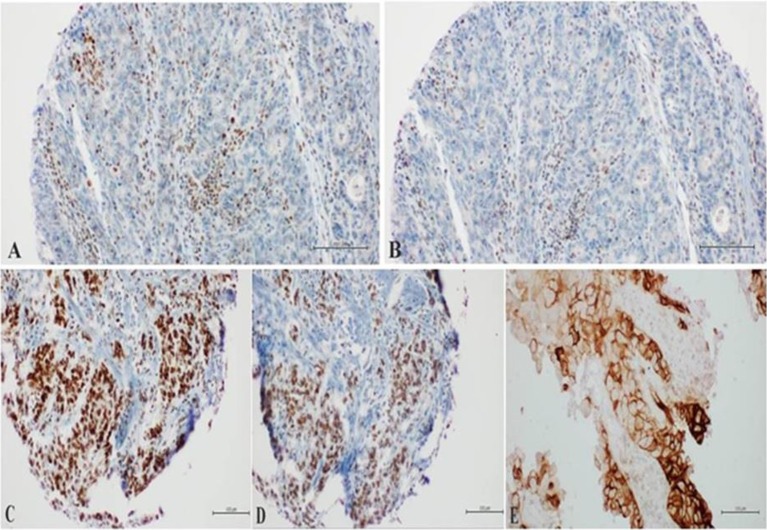
**(A,B)** Loss of nuclear staining of MLH1/PMS2 heterodimer in neoplastic cells with nuclear positivity in the Tumor-Infiltrating Lymphocytes (Tils); **(C,D)** preserved nuclear staining of MSH2/MSH6 heterodimer in neoplastic cells; **(E)** expression of PD-Ll in the neoplastic cells with Moderate to Intense circumferential membrane staining (10%).

After about 2 months of interruption, patient resumed Nivolumab in August 2018 and performed subsequently a new PET and CT scan (with contrast) in October 2018, which confirmed the complete radiological response (Figure 2, Figure S3). Meanwhile a thyroid function deterioration occurred and was corrected with the increase of hormone replacement therapy. Patient still maintained good clinical conditions and was asymptomatic. An abdominal MRI with contrast was performed in December 2018 in order to confirm the complete response (showing a millimetric possible necrotic residue). At the same time a primary tumor sample was submitted for FoundationOne CDx, which revealed high microsatellite instability (MSI-H), a high TMB of 58 mutations per DNA mega base, and several additional genomic alterations (Table S2). Furthermore, PD-L1 expression was 10% (tumor proportion score) and immunohistochemical mismatch repair testing showed loss of MLH1 and PMS2 heterodimer (Figure 3).

It is noteworthy that MLH1 promoter methylation analysis could be performed in order to explain the not mutation-caused changes in the protein functions (19).

Patient continued treatment and a further PET and CT evaluation confirmed no evidence of disease in April 2019 with no new onset relevant symptoms or toxicity after more than a total of 25 cycles of immunotherapy.

### Patient 2

An 82 year-old Caucasian male presented with dyspepsia and was found to have a poorly differentiated adenocarcinoma of the gastroesophageal junction (GEJ) (Lauren type unknown) causing 70% luminal obstruction. He was in good clinical condition (ECOG performance status 0); relevant comorbidities were blood hypertension, allergic rhinitis and cataract.

Staging CT scan confirmed a large mass of the GEJ with no evidence of distant disease (clinical stage III).

He subsequently underwent total gastrectomy with roux-en-y esophagojejunostomy and D2 lymphadenectomy in October 2015. The stage according to TNM was pT4a G3 pN3a (9+/16) cM0 HER2 negative (0 IHC score).

From December 2015 to May 2016, he received adjuvant chemotherapy with mFOLFOX-6 with modified dose due age and passed to De Gramont regimen at seventh cycle because of hematological toxicity.

In July 2016, PET and CT scan demonstrated a liver (single 37 mm lesion at VIII segment) and abdominal nodes recurrence for which patient started the second line treatment with paclitaxel 80 mg/mq (d1,8,5) plus ramucirumab 8 mg/kg (d1,15) administered in a 28 days cycle.

He was still in good clinical condition (ECOG performance status 0) with the exception of a slight weight loss and sporadic episodes of abdominal pain. He received 6 cycles with no grade 3–4 toxicity and achieved after the third cycle a partial response.

In March 2017 (PFS = 7 months), treatment was interrupted due to further liver disease progression with volumetric increase (35 × 32 mm) of the known lesion as confirmed by abdominal MRI with contrast (with no evidence of new lesions).

Because of the age and the unavailability of standard additional therapeutic options, patient was referred for stereotactic radiotherapy (30 Gy on liver and 30 Gy on abdominal nodes, respectively, in April and May 2017) and subsequently continued with radiological and clinical follow up until further liver (same VIII segment lesion) and nodal progression diagnosed at the CT scan performed 4 months later in September 2017 (with concomitant high blood levels of carcinoembryonic antigen CEA: 230 ng/mL).

On October 2017, 16th, patient started treatment with Nivolumab off label. At baseline the CT scan demonstrated a 39 mm liver lesion and several abdominal lymph nodes.

In December 2017, he performed, due to abdominal pain, a first CT scan evaluation, which showed stable disease according to RECIST criteria; he was still in good clinical condition with no immune-related toxicities.

After 4 months of treatment, he experienced episodes of pruritus without skin rash treated with oral cetirizine.

In April 2018, CT scan revealed a minor response both on liver and lymph nodes (with a normalization of blood levels of CEA: 3.5 ng/mL), while subsequently in September 2018 no evidence of disease was demonstrated with a completely negative evaluation (Figure 2, Figure S2).

**Figure 4:**
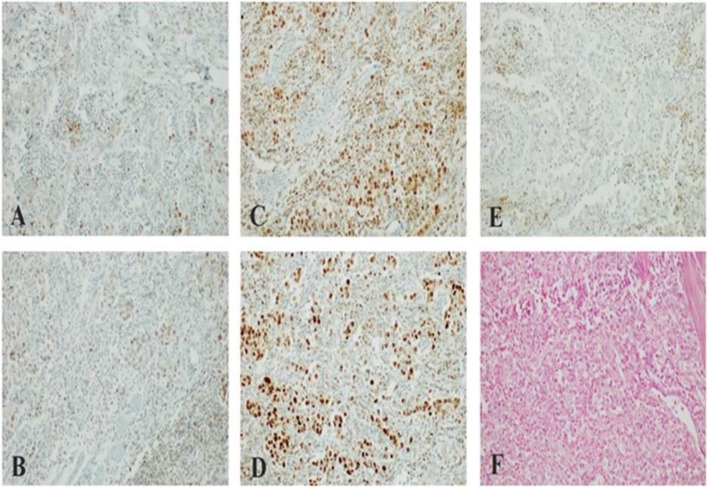
**(A,B)** “Patchy” nuclear staining of MLH1/PMS2 heterodimer in large neoplastic cells with scattering nuclear positivity of Tumor-Infiltrating Lymphocytes (TILs); **(C,D)** preserved nuclear staining of MSH2/MSH6 heterodimer in neoplastic cells; **(E)** lack of membranous staining of PD-Ll in large neoplastic cells with scattering positivity of Tumor-Infiltrating Lymphocytes (TILs); **(F)** lack of nuclear staining of Epstein-Barr virus (EBV)-encoded small RNAs (EBERs) (Patient 2).

Patient refused to undergo Foundation One CDx test, but local laboratory analysis on primary tumor sample revealed a so-called “patchy” expression pattern of MLH1 and PMS2 (interpretable as a plausible MMR deficiency although not a full loss of expression) and PD-L1 negative status (Figure 4). Moreover, EBER ISH for EBV status assessment has been performed with negative finding.

In January 2019 and May 2019, further CT scans confirmed complete radiological response and patient has been continuing immunotherapy with no new immune-related adverse events and good clinical condition after more than 30 cycles.

## Discussion

Precision medicine has largely remained elusive in GC to date. Immunotherapy represents a promising weapon although it is not yet a standard of care and the majority of evidence is limited to Asian population. The effectiveness of checkpoint inhibitors remains controversial in different settings of treatment.

Furthermore, the correct molecular patient selection is still debated and few data are available for Western patients although Pembrolizumab received US Food and Drug Administration approval across gastric tumors with PD-L1 positivity [as well as for MSI-H solid tumors in 2017 (20)]. The phase III trials available do not focus on the role of MSI and TMB as positive predictive factors.

Molecular and genomic differences between Asian and non-Asian GC, and particularly ethnicity-related differences in tumor immunity, have been already demonstrated and it is noteworthy that the only positive phase III trial with immunotherapy for GC is fully Asian to date (ATTRACTION-02).

Nevertheless, our report presents one of the first complete radiological responses to immunotherapy ever described to our knowledge in Western GC patients and the first ever to Nivolumab, considering the results of trials available to date.

Although only two cases are reported (with indolent course and low disease burden), we suggest that immunotherapy is promising also for non-Asian patients, and MSI testing and TMB are reliable and important also in this population despite the lack of evidence at current time.

Immunotherapy led to a real “disruption” of the prognosis and the natural history of these patients.

More data are expected for this “orphan” population and dedicated phase III trials, with a correct molecular patient selection, are urgently needed for this specific setting also in Western patients, despite the failure of previous line immunotherapy studies.

A further reflection regards the great benefit and good tolerability that also elderly patient (over 75 years old) can experience with immunotherapy and the results achievable thanks to a correct *continuum of care* strategy.

## Data Availability Statement

The raw data supporting the conclusions of this article will be made available by the authors, without undue reservation, to any qualified researcher.

## Ethics Statement

The studies involving human participants were reviewed and approved by Comitato Etico AOU Università degli Studi della Campania Luigi Vanvitelli—AORN Ospedali dei Colli, Via Santa Maria di Costantinopoli no 104, 80138 Naples. The patients/participants provided their written informed consent to participate in this study.

## Author Contributions

FD and GT designed the general outline of the paper supervising the writing of the paper. GT and APe wrote the main part and contributed to the clinical documentation collection and Supplementary Material selection. LP contributed to the clinical documentation collection and the literature review. APa and ML did the literature review and contributed to the English revision. IP, RS, and RF performed the molecular and histological analysis and created the corresponding images and figures. GG and FC critically contributed to the final review and the supervision giving the expert opinion. GT, APa, and LP also clinically followed during the years the patients treatments.

### Conflict of Interest

FC declares the following interests: Advisory Boards (Roche, Amgen, Merck, Pfizer, Sanofi, Bayer, Servier, BMS, Celgene, Lilly) and Institutional research grants (Bayer, Roche, Merck, Amgen, AstraZeneca, Takeda). FD declares the following interests: Advisory Boards (Roche, Amgen, Cellgene, Lilly). GT declares the following interests: Travel grant (Servier, Italfarmaco), Advisory (Lilly). The remaining authors declare that the research was conducted in the absence of any commercial or financial relationships that could be construed as a potential conflict of interest.
