# Supplementary material for: Durable Complete Radiological Response to Nivolumab in Two Heavily Pretreated Western Elderly Patients With Metastatic Gastric Cancer: A Case Report
Source: Front Oncol. 2020 Feb 17;10:130. doi: 10.3389/fonc.2020.00130 (PMC7039182; doi:10.3389/fonc.2020.00130)

**APPENDIX**

**TABLE OF CONTENTS**

**Table S1 (Immunohistochemistry/ISH panels and methods)..................................................Pag.2**

**Table S2 (Genomic Alterations FoundationOne Pt.1).............................................................Pag.3**

**Table S3 (List of abbreviations)..............................................................................................Pag.4**

**Figure S1 (MRI scan Pt.1)......................................................................................................Pag.5**

**Figure S2 (CT scan Pt2.)........................................................................................................Pag.6**

**Figure S3 (CT scan bis Pt.1)...................................................................................................Pag.6**

**
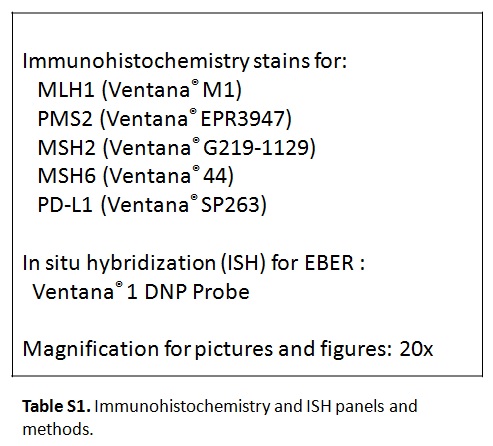
**

**
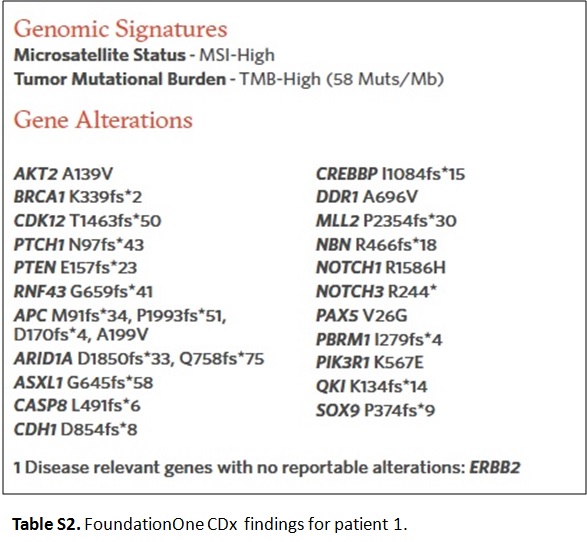
**


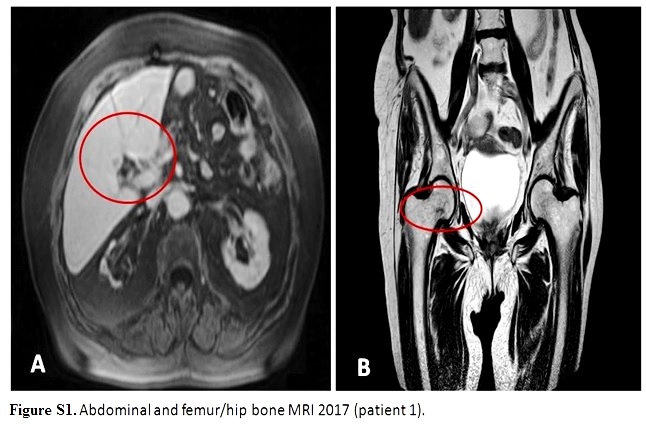


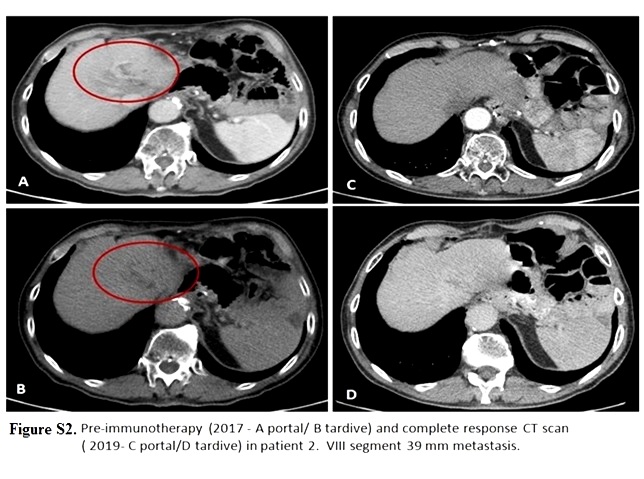

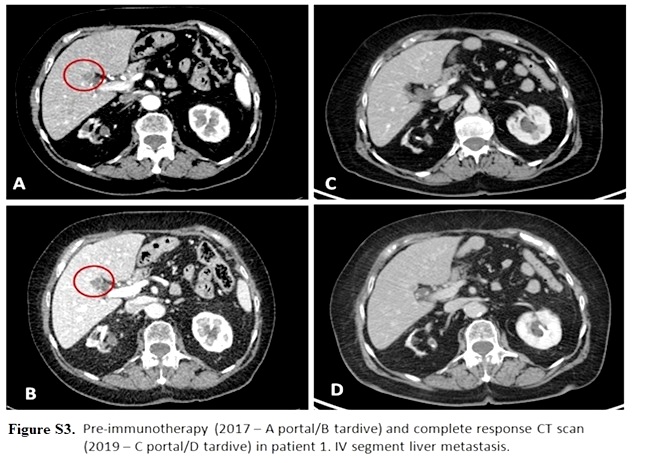

Supplement: Supplementary file 1 [file Table_1.DOCX]
